# Advanced Lipidomics in the Modern Meat Industry: Quality Traceability, Processing Requirement, and Health Concerns

**DOI:** 10.3389/fnut.2022.925846

**Published:** 2022-05-27

**Authors:** Chengliang Li, Burcu Ozturk-Kerimoglu, Lichao He, Min Zhang, Jiajing Pan, Yuanyi Liu, Yan Zhang, Shanfeng Huang, Yue Wu, Guofeng Jin

**Affiliations:** ^1^School of Food and Health, Beijing Technology and Business University, Beijing, China; ^2^Ege University, Engineering Faculty, Food Engineering Department, İzmir, Turkey; ^3^College of Food Science and Technology, Huazhong Agricultural University, Wuhan, China; ^4^School of Biology and Food Engineering, Chuzhou University, Chuzhou, China; ^5^Sonochemistry Group, School of Chemistry, The University of Melbourne, Parkville, VIC, Australia

**Keywords:** meat lipidomics, mass spectrometry, nuclear magnetic resonance, lipid biomarkers, lipolysis, biosynthesis, meat processing, nutritional value

## Abstract

Over the latest decade, lipidomics has been extensively developed to give robust strength to the qualitative and quantitative information of lipid molecules derived from physiological animal tissues and edible muscle foods. The main lipidomics analytical platforms include mass spectrometry (MS) and nuclear magnetic resonance (NMR), where MS-based approaches [e.g., “shotgun lipidomics,” ultra-performance liquid chromatography-tandem mass spectrometry (UPLC-MS/MS), matrix-assisted laser desorption and ionization time-of-flight mass spectrometry (MALDI-TOF-MS)] have been widely used due to their good sensitivity, high availability, and accuracy in identification/quantification of basal lipid profiles in complex biological point of view. However, each method has limitations for lipid-species [e.g., fatty acids, triglycerides (TGs), and phospholipids (PLs)] analysis, and necessitating the extension of effective chemometric-resolved modeling and novel bioinformatic strategies toward molecular insights into alterations in the metabolic pathway. This review summarized the latest research advances regarding the application of advanced lipidomics in muscle origin and meat processing. We concisely highlighted and presented how the biosynthesis and decomposition of muscle-derived lipid molecules can be tailored by intrinsic characteristics during meat production (i.e., muscle type, breed, feeding, and freshness). Meanwhile, the consequences of some crucial hurdle techniques from both thermal/non-thermal perspectives were also discussed, as well as the role of salting/fermentation behaviors in *postmortem* lipid biotransformation. Finally, we proposed the inter-relationship between potential/putative lipid biomarkers in representative physiological muscles and processed meats, their metabolism accessibility, general nutritional uptake, and potency on human health.

## Introduction

The global meat industry is a continuously growing sector with an ever-increasing demand. The Organization for Economic Co-operation and Development (OECD) and the Food and Agriculture Organization of the United Nations (FAO) have recently stated that worldwide meat production is projected to expand by approximately 44 million tons by 2030, despite the detrimental impacts of the coronavirus disease 2019 (COVID-19) pandemic and other possible restrictions (1). Meat is considered a unique animal-derived food providing high biological value proteins with all essential amino acids and various micronutrients (2). Alongside proteins, lipids are abundant constituents in meat and meat products that play critical roles in providing desirable mouth-feel perception, characteristic flavor, favorable texture, juiciness, and enhanced cooking yield (3). According to the complexity of structure and biosynthesis, lipids are divided into eight categories, depending on their differences in the level of unsaturation, the type of the covalent bond, the fatty acyl chain length, double bond location, the head groups, Z/E geometric isomerism, and the branched functional groups (4, 5). Specifically, these muscle-derived lipid species mainly include non-esterified/free fatty acids (FFAs), glycerolipids (GLs), glycerophospholipids (GPs), sphingolipids (SLs), sterol lipids, prenol lipids, saccharolipids, and polyketides (6). Among them, triglycerides (TGs) or triacylglycerols (TAGs) and phospholipids (PLs), two common and most abundant categories of lipids, are highly associated with health and nutritional functions in the body (7, 8). TGs are mainly composed of FAs, such as capric acid (Ca) and lauric acid (La), but their contents can vary greatly among different breeds and muscle tissues (9). Membrane phospholipid composition may play a critical role in subsequent lipid oxidation development in raw and cooked meats (10, 11), while SLs and glycolipids contribute more functions to human health, such as increasing anti-inflammatory and anticarcinogenic activities and alleviating the risk of cardiovascular diseases and cholesterol absorption (7, 12). Traditional studies utilize gas chromatography (GC) with known standards or mass spectrometry (MS) to detect and identify total fatty acids in the samples, where saponification and derivatization protocols are usually required to generate the volatile fatty acid analytes and may result in the loss of information about the original esterified structure (neutral or polar lipids) (9, 13). Thus, the modern meat industry deserves the exploration of lipid composition from different biological sources through global profiling for the qualitative and quantitative characterization of individual lipid species (11).

During the latest decade, scientific expertise and technologies are constantly being developed with commercial or industrial aspects to advance the traceability and authentication of meat products and to address the safety concerns of the public, and for economic and quality reasons as well (14). Although meat from different species can be easily detected using deoxyribonucleic acid (DNA)-based techniques, the mixing of meat from different biological sources (e.g., geographical origins) is more difficult to detect. In this regard, MS- and nuclear magnetic resonance (NMR)-based lipidomics, such as both untargeted and targeted approaches, have been suggested as a promising strategy for this detection (15–17). The high-throughput untargeted analysis has the advantage of detecting lipid metabolites as comprehensively as possible by emphasizing the changes in quantity in biological importance (14, 15). Additionally, the targeted approach focuses on identifying and acquiring a number of specific fractions of known lipid species, for instance, FFAs, PLs, and cholesterol, in the presence of external chemical standards or available databases (18, 19). To achieve more persuasive results, the sample/lipid-extract preparation, chromatographic separation or direct-infusion MS (DI-MS, “shotgun lipidomics”), method validation, multivariate data processing, and bioinformatics evaluation have been well considered in the entire analytical platform (4, 15).

Exploratory analysis, classification/discriminant analysis, and regression analysis/prediction models have been proven to be useful in lipidomics data analysis. Apart from these chemometric tools or machine learning methods, however, bioinformatic validation should be crucial for determining the potential biomarkers of lipid species present in a biological system and providing molecular insights into alterations in energetic/metabolic pathways of lipid biosynthesis (4). Hence, in this short review, we examined the mainstream of recently published investigation available with discussions regarding the applications of MS-/NMR-based lipidomics in muscle foods, such as meat origin and adulteration identification, meat safety assessment, and dietary lipid nutrition during representative processing conditions and/or *in vitro* treatment.

## Meat Lipidomics in Traceability and Microbial Safety

Meat is originally skeletal muscles of livestock and thereby suffers from some factors in the livestock production system, mainly such as animal genetic/breed background, feeding types, geographical location, and environmental stress, particularly associated with spoilage developed by microbiological activity (20). As highlighted in literature evidence (Table 1), different efforts have been undertaken to achieve lipid-label validation depending on diagnostic and reliable features that can reflect the origins/authenticity of suspected muscles and their freshness status through the lipidomics strategy. The consequent lipid molecules that are inherently from muscle tissues or result from the metabolism of indigenous microorganisms vary among different species, such as beef (14, 18, 21–24), pork (24–27), sheep/goat (28), poultry (16), and marine products (29–32). To investigate the effect of genetic background, MALDI-TOF-MS and Phosphorus-31 NMR (^31^P NMR)-based lipidomics were successfully applied to capture the differences in fatty acid biosynthesis among German Simmental bulls fed with different diets. Consequently, TGs, phosphatidylethanolamines (PEs), phosphatidylcholine (PCs), phosphatidylinositol (PIs), cardiolipins (CLs), and cholesterol were identified as potential biomarkers (22). Furthermore, untargeted and targeted MS-based lipidomics based on the use of ultra-performance liquid chromatography (UPLC) coupled with high-resolution MS (HRMS) has shown good discriminative power between different species/breeds and feeding conditions toward extended global lipid information with appropriate multivariate data analysis (Table 1). A panel of lipids, particularly GLs [diacylglycerols (DAGs) and TAGs], lysophosphatidylcholines (LPCs), lysophosphatidylethanolamines (LPEs), and n-6 polyunsaturated fatty acids (PUFAs) have been screened out as specific markers for differentiation of animal diets (18, 21, 25, 28, 29). More intermuscular differences traceable properties in lipidomics can be evaluated as a function of geographical origin and for adulteration (Table 1). For instance, GLs [TGs and diglycerides (DGs)] and GPs were screened as main lipid biomarkers by principal component analysis (PCA) and partial least squares-discriminant analysis (PLS-DA) modeling through UPLC-Q-TOF-MS/MS approach for China’s domestic pork (from Tibetan, Jilin, and Sanmenxia black pigs), suggesting the difference in production systems, feeds and genetic backgrounds (26).

**TABLE 1:** Representative applications of MS-/NMR-based lipidomics in meat origin/adulteration identification, nutritional/microbial quality, and biological function.

Type of origin	Species and muscle tissues	Sample preparation/lipid extraction methods	Analytical techniques	Data processing	Identified lipids and potential biomarkers	Possible biological functions and/or bioinformatics evaluation	References
Breed	Cattle-yak, yak, and cattle (*Longissimus* *thoracis*)	Extraction with methyl tertbutyl ether/methanol/water (2.6:2.0:2.4, v/v/v) with reconstitution in acetonitrile solution containing 0.04% acetic acid	UPLC-QTrap-MS/MS, untargeted	ANOVA, PCA, OPLS-DA, VIP	Phospholipids containing long-chain PUFAs, PAs, PCs, PEs, as well as SMs, CARs, FFAs, LPCs, CERs, TGs, DGs, MGs, long-chain acylcarnitines	Difference in energy metabolism and lipid nutrition quality, biomarkers of β-oxidation of fatty acids using KEGG database and enrichment	Gu et al. (21)
	Luchuan and Duroc boar pigs (*Longissimus* muscle)	Extraction with 70% aqueous methanol 4°C overnight	UPLC-QTrap-MS/MS, untargeted	ANOVA, *t*-test, PCA, OPLS-DA, HCA, VIP	TGs, PCs, PEs, DGs, and CERs	Regulation of lipolysis in adipocytes, fat digestion and absorption, and cholesterol metabolism enriched by KEGG database	Zhang et al. (25)
Feeding condition	Sheep/goat (*Biceps femoris*)	Aqueous/organic extraction by methanol/water (1:1, v/v) solution, dichloromethane/methanol (3:1, v/v) with resuspension of the dried extracts in isopropanol/acetonitrile (9:1, v/v)	UPLC-Q-TOF-MS, untargeted	ANOVA, PCA, OPLS-DA, VIP, permutation test, SVM	Glycerolipids (DAGs, TAGs), PCs, LPCs, PEs and SMs, acylcarnitines, n-6 PUFAs (arachidonic acid)	Functional components of membrane bilayers, energy storage, nutritional and physiological properties	Wang et al. (28)
	Beef steers tissues (Duodenum, liver, subcutaneous adipose, and *Longissimus dorsi*)	Phospholipids and cholesterol extraction by Bligh and Dyer’s liquid–liquid extraction (LLE) method	UPLC-TQ-MS, targeted	FDR *p*-adjustment, *t*-test, ROC, Pearson’s correlation analysis	PCs, PEs, LPCs, LPEs, cholesterol	Linoleic/α-linolenic acids metabolism and biosynthesis, metabolic crossroad induced by gain-to-feed ratios	Artegoitia et al. (18)
	German Simmental bulls fed with different diets (*Longissimus* muscle)	Homogenization in chloroform/methanol (2:1, v/v) followed by lipid isolation. Dried lipids restabilized in 50 mM Tris buffer (pH 7.65)	TLC separation, MALDI-TOF MS, ^31^P NMR spectroscopy (242.88 MHz)	PSD experiment (precursor ions), spectra deconvolution	TAGs, PEs, PCs, PIs, CLs, cholesterol	Regulating fatty acid biosynthesis in beef cattle under different dietary regimes	Dannenberger et al. (22)
	Hepatopancreas of mud crab (*Scylla paramamosain*) fed with DHA/EPA diets	Extraction with dichloromethane/methanol (3:1, v:v) followed by resuspension in isopropanol/acetonitrile/H_2_O (2:1:1, v:v:v)	UPLC-Q-Exactive-Orbitrap-MS, untargeted	*t*-test, PCA, PLS-DA, HCA, VIP	PCs, PEs, PSs, PIs, LPCs, SMs, TGs, and FFAs	Association with fatty acid transport/deposition, β-oxidation, long-chain PUFAs (DHA) biosynthesis using GO analysis, KEGG pathway enrichment	Wang et al. (29)
Geographical origin	Beef (from United States (US), Japan and Australia)	Homogenization with chloroform/methanol (1:1, v/v) followed by Bligh and Dyer’s liquid–liquid extraction (LLE) method	UPLC-Orbitrap-MS, untargeted	ANOVA, PCA, PLS-DA, OPLS-DA, VIP, LOO-CV, jackknife confidence intervals test	PCs, PEs and n-6/n-3 FFAs, MUFAs and particularly n-3 PUFAs	Nutritional quality and differential human diet	Man et al. (14)
	China’s domestic pork (from Tibetan, Jilin and Sanmenxia black pigs)	Homogenization and extraction with isopropanol	UPLC-Q-TOF-MS/MS, untargeted	ANOVA, PCA, PLS-DA, VIP	Glycerolipids (TGs, DGs), glycerophospholipids, sterol lipids, sphingolipids, polyketides, fatty acyls and prenol lipids	Main causes possibly including the difference in production systems, feeds and genetic backgrounds	Mi et al. (26)
	Beef (from six countries including Argentina, Australia, Brazil, Canada, New Zealand and Uruguay)	Homogenization with Folch solution [chloroform/methanol (2:1, v/v)] followed by dryness and re-solubilization	LC-Q-TOF-MS (comparison between DuoSpray and DART ion source), untargeted	RSD examination, PCA, fold change with *p*-adjustment of ANOVA, SVM	Glyceride (monoglyceride, diglyceride and triglyceride), FFAs, PIs, PEs, LPEs, LPCs, CARs, SMs, NAEs	Some indicators involved in lipid compositions, such as isotopic ratios, animal growth, production systems (feeding and exposure to microbials)	Wang et al. (23)
Freshness or microbial diversity	Farmed Atlantic salmon (*Salmo salar L.*) during storage at 4°C for up to 15 days	Extraction with methanol/water (2:5, v/v) and methyl tertary butyl ether followed by resuspension in isopropanol:acetonitrile (1:1, v/v)	UPLC-Q-Exactive-MS, untargeted	PCA, *t*-test, fold change with *p*-adjustment of ANOVA	LPCs and PCs	KEGG pathway showed linoleic acid metabolism, arachidonic acid metabolism and glycerophospholipid metabolism. The increase in LPC (C17:0) and LPC (C18:0) could result from the hydrolysis of PC (18:4/16:1) as a major freshness index.	Chen et al. (30)
	Large yellow croaker (*Larimichthys crocea*) fillets affected by cold treatment (−1°C and −3°C) during up to 35 days of storage	Homogenization in chloroform/methanol (2:1, v/v) followed by resuspension with isopropanol	UPLC-Q-Exactive-Orbitrap-MS, untargeted	ANOVA, Pearson’s correlation analysis, PCA, PLS-DA, VIP	CERs, CLs, DGs, HexCer, LPCs, LPEs, PCs, PEs, PGs, PIs, PSs, SMs, and TGs	Autophagy-animal, glycerophospholipid metabolism, linoleic/α-linolenic metabolism, arachidonic acid metabolism, and glycosylphosphatidylinositol (GPI)-anchor biosynthesis enriched by KEGG database	Chen et al. (31)
	Muscle from *Ctenopharyngodon idellus* during room-temperature storage for 72 h	Homogenization with chloroform/methanol (2:1, v/v) followed by Bligh and Dyer’s liquid–liquid extraction (LLE) method, dryness and re-solubilization	ESI-MS/MS (shotgun lipidomics), untargeted	Peak intensity screening, signal-to-noise ratio (SN) optimization, monitoring of precursor ion scan	Tracking phospholipid profiling including PCs, PEs, PIs, PSs, SMs	Oxidation and hydrolysis were mentioned as the two main causes for the deterioration of phospholipid in fish muscle during storage. PE molecular species may result from the microbe bred in the muscle.	Wang and Zhang (32)
Adulteration	Pork meat (hindquarter) from live pigs conventionally butchered versus dead pigs butchered immediately after death from diseases/abnormalities	Dual-phase extraction by methyl tert–butyl ether/methanol/H_2_O system, lipids redissolved in acetonitrile/isopropanol/H_2_O (65:30:5, v/v/v)	UPLC–TripleTOF–MS/MS, UPLC-QTrap-MS/MS, untargeted/pseudotargeted	Welch *t*-test, PCA, and HCA	PCs and TGs	The lower PCs content in dead pork implied the conversion into other metabolites, such as PEth.	Cao et al. (27)
	Different grades of beef mince and pork mince purchased from a national retail outlet	Homogenization in chloroform/methanol (1:1, v/v), lyophilised lipids redissolved in chloroform/methanol/water (1:4:4, v/v)	UPLC-LTQ Orbitrap-MS, untargeted	Kruskal–Wallis ANOVA, PLS-DA, Spearman’s correlation analysis, VIP	CERs, sphingolipids, PGs, TGs	Ceramide in sphingolipids metabolism by KEGG pathway analysis. Excessive irradiation may increase the content of free fatty acids, particularly in pork.	Trivedi et al. (24)
	Adulterated turkey breast muscle with protein hydrolysates	Homogenization first in ice-cold methanol, then ice-cold chloroform and finally ice-cold water. Storage at 4°C overnight and dried matter resuspended in an aqueous solution containing 0.05% TSP	^1^H NMR Spectroscopy (400 MHz), untargeted	ANOVA, PCA	o-phosphocholine, myo-inositol	The possible mechanism of lipolysis due to myo-inositol deficiency	Wagner et al. (16)

*TSP, sodium trimethylsilyl-2,2,3,3-tetradeuteroproprionate; DHA, docosahexaenoic acid; EPA, eicosapentaenoic acid; GC-FID, capillary gas chromatography coupled with flameionization detection; UPLC-Q-TOF-MS, ultraperformance liquid chromatography-quadrupole, time-of-flight mass spectrometry; UPLC-TQ-MS, ultraperformance liquid chromatography-triple quadrupole mass spectrometry; UPLC-TripleTOF-MS, ultraperformance liquid chromatography-triple time-of flight mass spectrometry; UPLC-QTrap-MS/MS, ultraperformance liquid chromatography-hybrid triple quadrupole-linear ion trap, tandem mass spectrometry; UPLC-LTQ Orbitrap-MS, ultraperformance liquid chromatography-hybrid linear ion trap-orbitrap, mass spectrometry; UPLC-Orbitrap-MS, ultraperformance liquid chromatography coupled with high-resolution orbitrap mass spectrometry; UPLC-Q-Exactive-Orbitrap-MS, ultraperformance liquid chromatography coupled to quadrupole exactive orbitrap high resolution mass spectrometry; TLC, thin-layer chromatography; MALDI-TOF MS, matrix-assisted laser desorption and ionization time-of-flight mass spectrometry; DART, direct-analysis-in-real-time ionization; ESI-QTrap-MS/MS: direct-infusion electrospray ionization-hybrid triple quadrupole-linear ion trap tandem mass spectrometry; NMR, nuclear magnetic resonance spectroscopy; ANOVA, analysis of variance; PCA, principal component analysis; PLS-DA, partial least squares discriminant analysis; OPLS-DA, orthogonal partial least squares discriminant analysis; HCA, hierarchical cluster analysis; VIP, variable importance in projection; LOO-CV, leave-one-out cross-validation; SVM, support vector machine; FDR, fold discovered rate; ROC, receiver-operator characteristic curve analysis; PSD, post source decay; RSD, relative standard deviations; TGs, triglycerides; DGs, diglycerides; MGs, monoglycerides; DAGs, diacylglycerols; TAGs, triacylglycerols; PAs, phosphatidic acids; PCs, phosphatidylcholines; PEs, phosphatidylethanolamines; PGs, phosphatidylglycerols; PSs, phosphatidylserines; PIs, phosphatidylinositols; LPCs, lysophosphatidylcholines; LPEs, lysophosphatidylethanolamines; SMs, sphingomyelines; CERs, ceramides; CARs, carnitines; NAEs, N-acyl ethanol-amines; HexCer, hexosylceramide; CLs, cardiolipins; FFAs, free fatty acids; MUFAs, monounsaturated fatty acids; PUFAs, polyunsaturated fatty acids; PEth, phosphatidylethanol; FAMEs, fatty acid methyl esters; GO, gene Ontology; KEGG, service of Kyoto Encyclopedia of Genes and Genomes.*

It is also noticeable that exposure to microbes may significantly influence the lipid compositions during meat production where other indicators are usually involved, e.g., isotopic ratios and feeding (23). Particularly for marine products, such as fish, fatty acids, and GPs, metabolism is identified as the major pathway through microbial contamination during cutting, storage, and distribution processes after slaughter. A study was conducted to evaluate the spoilage of farmed Atlantic salmon (*Salmo salar L.*) during storage at 4°C for up to 15 days by adopting the UPLC-Q-Exactive-MS with high sensitivity (30). According to the results, the increase of LPC (C17:0) and LPC (C18:0) could result from the hydrolysis of PC (C18:4/C16:1) as a major freshness index. Assisted by “shotgun lipidomics,” PCs, PEs, PIs, phosphatidylserines (PSs), and sphingomyelins (SMs) were profiled as the lipid biomarkers of interest for the naturally spoiled muscle from *Ctenopharyngodon idellus* during room-temperature storage. UPLC-HRMS-based lipidomics has also shown good strength in identifying PCs, ceramides (CERs), and SLs metabolism as differentiated by pork and beef ground meat from different grades or due to death from diseases/abnormalities (24, 27). However, NMR-based lipidomics can screen more polar lipid metabolites and thus provides characteristic information on the metabolic profile of adulterated muscle. The presence of o-phosphocholine and a reduced level of Myo-inositol in turkey breast muscle injected with protein hydrolysates were observed through Proton NMR (^1^H NMR) untargeted lipidomics, suggesting the possible role of Myo-inositol deficiency in enhanced lipolysis (16, 33).

## Meat Lipidomics as Affected by Processing Factors

Up to date, various meat processing strategies, such as castration (34), thermal/non-thermal techniques (8, 17, 19, 35–38), freezing/thawing intervention (39), *in vitro* oxidation (40, 41), *postmortem* aging/storage (11, 42), modified atmosphere packaging (MAP) (42), and brining/drying-curing/preservatives treatments (43–47) have been implicated to improve the microbiological safety, color, flavor, and texture for the development of favorable meat products (Table 2). Accordingly, DI-MS and/or a combination of GC and liquid chromatography (GC/LC) and MS techniques have been utilized in the application of thermal processing to identify dozens of different lipids as potential biomarkers. Consequently, the lipolysis of TGs and PLs was noted as a strong flavor-binding precursors through different thermal degradation (boiling, steaming, and roasting) and showed specific losses of representative lipids (19, 35). It is worthwhile to note that fresh fish fillets, particularly *Pleuronectes platessa* upon high-pressure processing (HPP) were characterized by a distinctly high level of lipid-derived polar metabolites, serine-phosphoethanolamine species (Ser-PETA) (36). Specifically, studies on the discrimination of untreated/irradiated raw/ground meat from commercially produced goat, chicken, turkey, and pork through global lipid profile using UPLC-Q-Exactive-Orbitrap-MS/MS, targeted GC-resolved fatty acids composition, and chemometric tools (PCA and PLS-DA) have been recently reported (8, 37, 38). These authors observed γ-ray irradiation dose-dependent increase in docosahexaenoic acid (DHA)-enriched PC (C18:4/C22:6) + H in goat meat, while X-ray irradiation tended to result in n-3 PUFA-enriched lipids in chicken and turkey meat, accompanied with a noticeable accumulation of PLs and oxidized short- and long-chain FFAs. Comprehensive lipidomics based on GC-technique and phosphorus-31/carbon-13 NMR (^31^P/^13^C NMR) spectroscopy was devoted for hoki co-product (i.e., roe) treated with a pulsed electric field (PEF), where abundant PLs and a high level of lyso-diphosphatidylglycerols (LDPGs), LPEs, lysophosphatidylserines (LPSs), and LPCs were finally characterized, and PEF transformed more *sn*-2 phospholipid eicosapentaenoic acid (EPA) and DHA into *sn*-1,3 positions with potentially compromised bioavailability in terms of re-esterified structures (17). Additionally, when frozen Atlantic salmon and bullet tuna were thawed, an untargeted MS-based lipidomics approach detected abundant water-soluble phospholipid metabolites (i.e., L-α-glyceryl-phosphoryl-choline and *N*-methyl-ethanolamine phosphate) (39). However, hydroxyl radical (^⋅^OH) attacks can significantly alter the lipidomics profiles of shrimp and yak muscle to a large extent. PEs enriched in PUFAs were highly vulnerable to *in vitro* oxidation but both GPs metabolism and fatty acid biosynthesis were enriched during the subsequent deterioration and spoilage process of oxidized yak hindquarter meat (40, 41). On the other hand, PLs in porcine meat can undergo enzymatic hydrolysis during *postmortem* aging. For example, by employing targeted UPLC-TQ-MS/MS (with PLs as internal standards) to determine the PLs lipolysis tendency, researchers found that *postmortem* porcine loin (*M. Longissimus*) up to 21 days at 4°C was rich in PIs, PSs, and PAs, particularly C38:4 and C36:2 lipid species. Phospholipase A_2_ (PLA_2_) can be activated by *postmortem* calcium influx from the sarcoplasmic reticulum (SR) with a surge of LPCs in aging muscles (11). Apart from the targeted MS approach, untargeted “shotgun lipidomics” [electrospray ionization (ESI)-QTrap-MS/MS] became useful in identifying the lipid biomarkers derived from water-boiled dry-cured Pekin duck as a function of salting and ripening times (43, 44). In those studies, the findings pointed out that low-salt (< 6%) dry-cured duck significantly promoted the degradation of individual PLs (e.g., PCs, PGs, PEs, PSs, and PIs) probably resulting from a robust release of phospholipase, though some LPCs were damaged possibly due to oxidation and thermal degradation provided by boiling. In other cases, by combining the GC-system and untargeted hydrophilic interaction liquid chromatography (HILIC) coupled to QTrap-MS, saturated fatty acids (SFAs) (C16:0), monounsaturated fatty acids (MUFAs) (C18:1), and PUFAs (C18:2) were observed to be important lipid-derived flavor precursors in low-salted salmon and PCs content was kept at a high level even at 30% NaCl replacement rather than PSs (45). Following untargeted UPLC-QTrap-MS/MS lipidomics, salting/preservatives treatment of goat meat led to decrements in TGs concentration (47) while dry-curing of mutton ham (M. *biceps femoris*) significantly contributed to GLs, DGs, and specifically C20:3 and C18:4 FFAs released, showing characteristic metabolisms of GPs and SLs (46).

**TABLE 2:** The implications of MS-/NMR-based lipidomics in processed meat, quality control, and metabolism monitoring.

Processing factors	Species and muscle tissues and/or co-product	Sample preparation/lipid or metabolites extraction methods	Analytical techniques	Data processing	Identified lipids and potential biomarkers	Main results/possible biological functions and/or bioinformatics evaluation	References
Castration	Psoas major muscle of lambs	Homogenization with chloroform/methanol (2:1, v/v) followed by liquid–liquid extraction (LLE), dryness, and resuspension in chloroform/methanol (2:1, v/v)	UPLC-Q-Exactive-Orbitrap-MS/MS, untargeted	ANOVA, Student’s two-tailed *t*-test, PCA, HCA	Major lipid species identified as PCs, PEs, SMs, TGs, FFAs, DGs, particularly in the castration group	Castration could increase IMF content and modify the intramuscular TGs/phospholipids ratio and the PUFAs/SFAs ratio.	Li et al. (34)
Thermal processing	Boiled, steamed and roasted Tan sheep meat (M. *longissimus dorsi*)	Lipid extraction with 100% isopropanol alcohol followed by protein precipitation, centrifugation, and collection of the supernanant	UPLC-Q-Exactive-Orbitrap-MS/MS, untargeted	p-adjustment, RSD examination, PCA, PLS-DA, VIP, HCA	SMs, CERs, LPCs, PCs, PEs, TAGs	The boiled approach was representative of more losses of SMs than CERs in meat, while the steamed one contributes to losses of PCs and LPCs in glycerophospholipid metabolism. These processed diets provided different options to the patients with atherosclerosis and cancer, the elderly, and infants.	Jia et al. (35)
	Roasted mutton (M. *back strap*) from 6-month-old sheep	Fatty acids in lipids extracted with dichloromethane/methanol solution (2:1, v/v) followed by phase-separation, restabilized in butylated hydroxytoluene/ hexane (0.02%, w: v) and methylation. Lipid extraction for lipidome analysis using isopropanol followed by centrifugation, and collection of the supernanant.	GC-FID, targeted (with FAMEs external standards); UPLC-Q-TOF-MS/MS, untargeted	ANOVA, OPLS-DA, VIP, Correlation analysis	TGs (e.g., C16:0/C18:1/C18:1, C18:0/C18:0/C18:1), PCs (C30:6, C28:3), and PEs. FFAs such as C16:0, C18:0 and C18:1.	TGs should be predominant lipids relevant to the aroma binding stability during roasting times. Phospholipids content showed a negative correlation with characteristic aroma, e.g., pentanal, hexanal, and heptanal, suggesting lipolysis and oxidative degradation.	Liu et al. (19)
High pressure processing (HPP)	Fresh fish fillets (*Salmo salar* and *Pleuronectes platessa*)	Homogenization with perchloric acid (0.1 mol/L) to extract polar metabolites followed by centrifugation and supernatant separation	UPLC-Q-Exactive-Orbitrap-MS/MS, untargeted	ANOVA, HCA, Volcano Plot (VP)	High concentration of lipid-derived serine-phosphoethanolamine species (Ser-PETA), particularly in *Pleuronectes platessa* group regardless of HPP	Some specific polar lipid-metabolites (e.g., Ser-PETA) might be active in fatty acid transformation and participate in glycerophospholipid metabolism.	Castrica et al. (36)
Pulsed electric field (PEF)	Hoki roe treated with PEF at different field strengths (0.62, 1.25, 1.875 kV/cm) and frequencies (25, 50, 100 Hz)	Total lipid extracted with hexane/methanol (1:2, v/v) using ETHEX partition method followed by homogenization, filtration and evaporation	GC-FID, targeted (with FAMEs external standards); ^31^P NMR spectroscopy (162 MHz, to analyze phospholipid composition), ^13^C NMR spectroscopy (100 MHz, to analyze the ratio of positional distribution of EPA and DHA on TAGs)	Semi-quantification in the abundance of each lipid using the integrated response of the NMR spectra, two-way and one-way ANOVA	PAs, PEs, PSs, PIs, PCs, LDPGs, LPEs, LPSs, LPCs, CLs, and SMs; n-3 fatty acids, i.e., DHA/EPA esterified at *sn*-2/*sn*-1,3	High PEF input resulted in abundant phospholipids without affecting n-3 fatty acid content, and generated LDPGs, LPEs, LPSs and LPCs. PEF transformed more *sn*-2 phospholipid EPA and DHA into *sn*-1,3 positions, indicating a negative change in re-esterified structures to phospholipids and decreased bioavailability of hoki roe lipids.	Ahmmed et al. (17)
γ-ray irradiation	Goat meat (uncastrated, from *Longissimus dorsi*) irradiated at different doses (0, 1, 2, 4 and 6 kGy)	Extraction with methanol and MTBE, dried lipids restabilized in acetonitrile/isopropanol/H_2_O (65:30:5, v/v/v)	UPLC-Q-Exactive-Orbitrap-MS/MS, untargeted	RSD examination, ANOVA, PCA, PLS-DA, VIP	Increased content in TGs, PCs, PEs, LPEs, CERs, LPCs and SPHs; decreased level of DGs, PSs, PGs, PIs and SMs after irradiation	Lipid variables were involved in the major pathways of glycerophospholipid and sphingolipid metabolism. DHA-enriched PC (C18:4/C22:6) + H exhibit an increase upon irradiation.	Jia et al. (37)
X-ray irradiation	Chicken, turkey and mixed (chicken, turkey and pork) ground meat irradiated at different doses (0, 0.5, 1, 3 and 5 kGy)	Homogenization with chloroform/methanol (1:2, v/v) followed by Bligh and Dyer’s liquid–liquid extraction (LLE) and resuspension in 95% hexane and dryness. Additional methylation for the extracted fatty acids.	GC-FID, targeted (with FAMEs external standards); UPLC-Q-Exactive-Orbitrap-MS/MS, untargeted	ANOVA, PCA, Volcano Plot (VP)	DGs, TGs, SMs, CERs, LPGs, LPIs, LPEs, LPCs, PIs, PEs, PCs. PSs, and PGs. Phospholipids increased in a dose dependent manner with enriched level of PUFAs	The content of n-3 PUFA-enriched lipids in irradiated chicken and turkey meat reflected the meat’s nutritional value. Oxidized phospholipids (OxPLs) were identified in low abundance as a potential new biomarker.	Chiesa et al. (8)
	Chicken, turkey and mixed (chicken, turkey and pork) ground meat irradiated at different doses (0, 0.5, 1, 3 and 5 kGy)	Extraction with cold mixture (0.1% formic acid, H_2_O/methanol (20:80, v/v)	UPLC-Q-Exactive-Orbitrap-MS/MS, untargeted	ANOVA, HCA, Box-Whisker charts (BWC), Volcano Plot (VP), paired *t*-test	Short and long-chain fatty acids (e.g., oxidized *cis*, *cis*-1,4-pentadiene fatty acids)	PUFA and their oxidative derivatives should be good biomarkers to speculate lipid oxidation pathway in ground meat triggered by irradiation	Panseri et al. (38)
Freezing/thawing processing	Atlantic salmon (*Salmo salar*) and bullet tuna (*Auxis rochei*) during freeze (−20°C/−35°C and −18°C, respectively)/thaw processing	Homogenization with perchloric acid (0.1 M) to extract polar metabolites followed by centrifugation, supernatant collection and dilution	UPLC-Q-Exactive-Orbitrap-MS/MS, untargeted	ANOVA, fold change with p-adjustment, PCA, Volcano Plot (VP), Box-Whisker charts (BWC) with descriptive statistics	L-α-glyceryl-phosphoryl-choline, *N*-methyl-ethanolamine phosphate	These two water-soluble phospholipid metabolites increase upon thawing of frozen samples regardless of the storage period, suggesting an impaired phospholipid membrane integrity, and enhanced phospholipid catabolism and lipid oxidation	Chiesa et al. (39)
*In vitro* oxidation	Whiteleg shrimp (M. *Litopenaeus vannamei*) upon H_2_O_2_/ascorbate-based hydroxyl radical (^⋅^OH)-generating system (1, 2 and 4 mM H_2_O_2_)	Homogenization with cold chloroform/methanol (2:1, v/v) followed by liquid–liquid extraction (LLE), concentration, and resuspension in isopropanol	UPLC-TripleTOF-MS/MS, untargeted	ANOVA, PCA, OPLS-DA, permutation test, VIP	PCs (C38:3), CLs (C62:2), and PEs (C34:9)	Hydroxyl radical attack can alter the lipidomics profiles of shrimp muscle to a large extent. High concentration of oxidizing conditions exacerbated lipid peroxidation. PEs enriched in PUFAs are more susceptible to oxidation *via* radical attack than PCs molecules.	Tu et al. (40)
	Yak hindquarter meat in a Fenton oxidation system (FeCl_3_/ascorbate/10 mM H_2_O_2_) followed by refrigerated storage at 4°C for 2 h	Tissue extract mixed with cold 75% chloroform/methanol (1:9, v/v) and 25% H_2_O by two-step extraction toward metabolite optimal recovery	UPLC-Q-Exactive-Orbitrap-MS/MS, untargeted	ANOVA, PCA, PLS-DA, OPLS-DA, HCA	FFAs (e.g., stearic acid, linoleic acid, arachidonic acid)	Notable glycerophospholipid metabolism in oxidized yak meat indicated deterioration and spoilage process. 2-hydroxy-3-oxoadipate was assumed to promote fatty acid oxidation, while arachidonic acid could be involved in fatty acid biosynthesis as revealed by KEGG enrichment	Huang et al. (41)
*Postmortem* aging/Packaging/Storage	*Postmortem* porcine loin (Longissimus) during aging for 1, 8 and 21 days at 4°C	Homogenization with chloroform/methanol (1:1, v/v) followed by liquid–liquid extraction (LLE), dryness, and resuspension in chloroform	UPLC-TQ-MS/MS, targeted (with phospholipid internal standards)	ANOVA, Tukey and Tukey-Kramer *p*-adjustments	PIs (e.g., C38:4), PSs (e.g., C36:2), LPCs, and PAs	Phospholipids underwent enzymatic hydrolysis during aging (except for C18:2 or C20:4 within PI and PS). Phospholipase A2 (PLA2) could be activated by postmortem calcium influx from the sarcoplasmic reticulum (SR) with a surge of LPCs	Chao, Donaldson et al. (11)
	*Postmortem* bovine muscle, M. *longissimus dorsi lumborum* by different packaging methods, i.e., under high oxygen modified atmosphere, oxygen permeable film, and vacuum-packaging during up to 14 days	Extraction of lipids by Folch’s method, homogenization with chloroform/methanol (2:1, v/v) followed by standing overnight at 4°C and separation of organic layer	MALDI-TOF-MS, MALDI–MSI, targeted (phospholipids, triglycerides and sterols)	Semi-quantification in the abundance of each marker lipid (with targeted m/z)	PCs, LPCs, PEs, LPEs, TAGs and sterols	PCs (except for C18:1/C18:0) were sensitive to oxidative degradation while cholesterol showed relatively high stability to oxidation.	Dyer et al. (42)
Salting/Drying-curing/Preservatives treatment	Water-boiled dry-cured Pekin duck at 6% saute-salt (w/w) following 3 days of ripening	Phospholipid extraction with chloroform/methanol (1:2, v/v) followed by Bligh and Dyer’s liquid–liquid extraction (LLE), centrifugation, dryness and dilution	ESI-QTrap-MS/MS (shotgun lipidomics), untargeted	ANOVA, PCA, PLS-DA, VIP	LPLs (C18:2), PEs, PCs [C34:2 (C16:0/C18:2)], PGs, PIs, and PSs	Processing decreased most of the phospholipid molecular species but increased LPLs content until extended ripening (2 days). Boiling resulted in loss in some of the LPLs suggesting an oxidative thermal-degradation and decomposition.	Li et al. (43)
	Water-boiled dry-cured Pekin duck with three different salt contents: 4% (low-salt), 6% (medium-salt) and 8% (high-salt)	Phospholipid extraction with chloroform/methanol (1:2, v/v) followed by liquid–liquid extraction (LLE), centrifugation, dryness and dilution	ESI-QTrap-MS/MS (shotgun lipidomics), untargeted	ANOVA, PLS-DA, VIP	PCs, PGs, PEs, PSs, and PIs	Low-salt (< 6%) dry-cured duck had a significant effect on total phospholipid content and promoted the degradation of individual phospholipids (especially those containing unsaturated fatty acids) probably due to a robust release of phospholipase.	Li et al. (44)
	Low-salted salmon treated with sodium replacers (KCl, CaCl_2_) and flavor enhancers (yeast extract, lysine, taurine)	Homogenization with Folch solution [chloroform/methanol (2:1, v/v)] followed by phase-separation and dryness. Methylation for the extracted fatty acids. Crude lipids re-stabilized in chloroform toward phospholipids analysis	GC-FID, targeted (with FAMEs external standards); HILIC-QTrap-MS, untargeted	ANOVA, *post hoc* Duncan multiple range tests, PCA, OPLS-DA, HCA, VIP	SFAs (C16:0), MUFAs (C18:1), PUFAs (C18:2), PCs, PEs, PSs, and PIs	PCs content remained high even at 30% NaCl replacement rather than PSs. The addition of flavor enhancers increased the total content of phospholipids.	Wang et al. (45)
	Dry-cured mutton ham (M. *biceps femoris*) from Xinjiang fine-wool sheep processed through 105 days of fermentation and 60 days of ripening	Lipid extraction with MTBE/methanol (3:1, v/v) followed by liquid–liquid extraction (LLE), centrifugation and concentration	UPLC-QTrap-MS/MS, untargeted	ANOVA, PCA, OPLS-DA, VIP, fold change with *p*-adjustment, permutation test, Volcano Plot (VP)	PCs, PEs, PSs, LPCs, and LPEs	FFAs content increased during ham processing. Glycerophospholipid metabolism and sphingolipid metabolism were mentioned as the most important metabolic pathways using KEGG database and MSEA analysis.	Guo et al. (46)
	Four treatments of Hengshan goat meat sausages (preservative-free, natamycin, potassium sorbate and sodium diacetate)	Lipid extraction with 100% isopropanol alcohol followed by protein precipitation, centrifugation and organic-phase separation	UPLC-Q-Exactive-Orbitrap-MS/MS, untargeted	RSD examination, fold change with *p*-adjustment, PCA, PLS-DA, VIP	CERs, DGs, LPCs, PCs, PEs, PIs, PSs, SMs, TGs	Preservative treatments decrease of TGs concentration in goat meat. Significant lipid variables are related to glycerophospholipid, and sphingolipid metabolism as explained by KEGG pathway.	Jia et al. (47)

*MTBE, methyl tert-butyl ether; ETHEX, ethanol and hexane for lipid extraction; DHA, docosahexaenoic acid; EPA, eicosapentaenoic acid; GC-FID, capillary gas chromatography coupled with flameionization detection; UPLC-TQ-MS/MS, ultraperformance liquid chromatography-triple quadrupole tandem mass spectrometry; HILIC-QTrap-MS, hydrophilic interaction liquid chromatography-hybrid triple quadrupole-linear ion trap, mass spectrometry; ESI-QTrap-MS/MS, electrospray ionization-hybrid triple quadrupole-linear ion trap, tandem mass spectrometry; UPLC-QTrap-MS/MS, ultraperformance liquid chromatography-hybrid triple quadrupole-linear ion trap, tandem mass spectrometry; UPLC-Q-Exactive-Orbitrap-MS/MS, ultraperformance liquid chromatography coupled to quadrupole exactive orbitrap high resolution tandem mass spectrometry; UPLC-TripleTOF-MS/MS, ultraperformance liquid chromatography-triple time-of flight tandem mass spectrometry; MALDI-TOF MS, matrix-assisted laser desorption and ionization time-of-flight mass spectrometry; MALDI-MSI, matrix-assisted laser desorption/ionization mass spectrometric imaging; NMR, nuclear magnetic resonance spectroscopy; ANOVA, analysis of variance; PCA, principal component analysis; PLS-DA, partial least squares discriminant analysis; OPLS-DA, orthogonal partial least squares discriminant analysis; HCA, hierarchical cluster analysis; VIP, variable importance in projection; RSD, relative standard deviations; TGs, triglycerides; DGs, diglycerides; TAGs, triacylglycerols; PAs, phosphatidic acids; PCs, phosphatidylcholines; PEs, phosphatidylethanolamines; PGs, phosphatidylglycerols; PSs, phosphatidylserines; PIs, phosphatidylinositols; LPGs, lysophosphatidylglycerols; LPCs, lysophosphatidylcholines; LPSs, lysophosphatidylserines; LPEs, lysophosphatidylethanolamines; LDPGs, lyso-diphosphatidylglycerols; CLs, cardiolipins; SMs, sphingomyelines; SPHs, sphingosine bases; CERs, ceramides; FFAs, free fatty acids; MUFAs, monounsaturated fatty acids; PUFAs, polyunsaturated fatty acids; SFAs, saturated fatty acids; FAMEs, fatty acid methyl esters; KEGG, service of Kyoto Encyclopedia of Genes and Genomes; MSEA, metabolite set enrichment analysis.*

## Discussion on Possible Mechanisms and Future Remarks

Lipids are inherently abundant in muscle tissues, which play critical roles in a series of cellular processes and physiological/biological activities (e.g., cell membrane architecture, cell signaling and energy-storing) (48). Breeds and feeding conditions, which usually have a large impact on animal physiology, determine the skeletal muscle growth and maturation, the final meat yield, and nutritional/flavor quality (21, 28, 29). NMR-/MS-based approaches have been attempted to obtain lipid-metabolite signatures and their relationship to physiological alteration in tissues as well as the potency in meat production. Differences in muscle energy metabolism, lipolysis in adipocytes, fat digestion/absorption, and cholesterol metabolism, β-oxidation of fatty acids, in particular, would offer the dynamics of redox status, oxidative stability, and consumer acceptability as influenced by the nutrients and ingredients in the animal feeds (20). For instance, concentrate-fed sheep are generally more obese compared with pasture-grazing sheep. High-fat diets may thereby promote the levels of isoleucine, lipids, glutamate, and 3-methylhistidine and lead to decreased citrate and glycerophosphorylcholines (GPCs) in animals (28). Consequently, as putatively revealed from lipidomics analysis, DAGs tend to accumulate in meat from concentrate-fed sheep/goats due to fat deposition, while some saturated TGs (e.g., C40:0, C42:0, and C44:0) in meats can be favored by pasture-grazing feeding strategy, showing a positive biological function of energy storage (28). PLs are the primary structural constituents of biological membranes and serve as critical nutrients owing to their physiological and nutritional properties. Following lipidomics, some species of fatty acid components and PLs have been useful markers in muscle tissues for differentiating breeds/dietary supplementation (21, 28, 29), detecting adulteration (27), and monitoring microbial accessibility (30–32). For instance, n-3 long-chain PUFA, such as EPA (C20:5n-3) and DHA (C22:6n-3) are essential fatty acids (EFA) for marine fish and crustaceans. An appropriate ratio of DHA/EPA feeding diet can improve the lipogenesis, integrity of membrane PLs, and DHA biosynthesis/deposition, meanwhile, inhibiting the mitochondrial β-oxidation of fatty acid and supporting growth performance in the hepatopancreas of *S. paramamosain* (29).

Lipids in the meat matrix are usually involved in thousands of metabolites that may be affected by species, nutrients, microbial diversity, production, and storage. These muscle-derived lipid metabolites are not only the phenotypic consequences of physiological muscle metabolism but also the major molecular basis for characterizing organoleptic components following different processing conditions (20, 49–51). For dry-cured meats, lipolysis usually involves a set of endogenous adipose tissue TGs lipases [e.g., neutral and basic ones including hormone-sensitive lipases (HSL) and lipoprotein lipases (LPL)] and some phospholipases (classified as A_1_, A_2_, C, and D) responsible for PLs degradation followed by auto-oxidation, which contributes to the formation of aromatic volatile compounds (10, 52, 53). However, we should note that the identification efficacy of bioactive lipid species through classical MS-based lipidomics approaches would be significantly affected by the applied lipid extraction protocols [e.g., liquid–liquid extraction (LLE), some alternative methods, such as methyl tert-butyl ether (MTBE)] mainly due to the characteristic amphipathic properties of lipids to achieve a differential partition (54, 55). In particular, the good recovery, ionization efficiency, and identification of global phospholipid species by LC-MS are still difficult to achieve, arising from their complexity in molecular structures (i.e., the length of the fatty acid chains and the difference in fatty acyl substitution at the glycerol backbone), and hydrophilicity across the entire chromatographic separation (28, 45, 56). Regarding the MS instruments showing high sensitivity, multi-sourced trace impurities that could result from biological matrices (e.g., remaining proteins in muscle tissues), solvents used for lipid extraction, preparation devices, such as siloxenes and phthalates, and even sample containers, such as plasticizers could be detected when they are carried to the lipid extract and thus these impurities may influence the reproducibility of the lipidomics profile (54). So, the extension of GC-system and NMR-based lipidomics and their combination with LC-resolved MS would exert unique superiority to enrich the entire lipid metabolism pathway (e.g., biosynthesis, oxidative decomposition, and enzymatic lipolysis) by detecting FAs, TGs, and sterols as well as some specific short and polar secondary lipid-metabolites (8, 16, 17, 19, 22). Indeed, during non-thermal processing, such as HPP, some important water-soluble lipid-metabolites (e.g., Ser-PETA) might be active in fatty acid transformation and participate in GPs metabolism (36). Overall, the lipolysis of TGs and PLs in meats are closely related to some relevant factors, mainly including (i) the feeding processes of animals (28, 29), (ii) circumstances in slaughtering (30, 31), (iii) *postmortem* aging (11, 42), (iv) thermal/non-thermal processing (8, 13, 17, 19, 35, 37, 57), and (v) brining/dry-ripening processes (45–47, 58). As a result, the physical/redox status of muscle/adipose tissues can be changed with the difference in bioavailability and bioactivity of endogenous and microbial lipases/phospholipases (10, 13, 52, 53, 59–63), consequently determining the fat deposition, lipolysis, and lipidomics profile (9, 20). In most cases, PLs are the main substrates for lipolysis in dry-cured meat products (10). However, some protein chaperones (heat shock protein 90, Hsp90) are reported to stabilize cell membranes and preserve membrane integrity in muscle tissues, and particularly act as an inherent antioxidant by providing additional protection against ROS-induced PLs oxidation (64). For seafood, such as in fish fillets, the total lipid content generally decreases during cold storage as TGs and PLs are either hydrolyzed by lipolytic enzymes, such as lipase and PLA_2_ and/or susceptible to oxidative damage from the myoglobin-mediated mechanism of action (39, 65, 66), though the activity of mitochondrial enzymes may be different during subsequent thawing. Indeed, the water-enriched external medium could induce hydrostatic pressure in cells and impair plasma membrane integrity, provoking an enrichment of intracellular enzymes in the final exudate (39). These knowledge of the adipose tissue TGs and PLs hydrolysis and oxidation during meat processing suggests a complicated overall lipid degradation mechanism and cellular protection under oxidative stress. A more comprehensive understanding based on multi-omics techniques is still required for improving both the quality and nutritional value of specific end products.

## Conclusion

During the transition from “farm-to-fork” to the modern meat industry, the lipidomics disciplines successfully encompass a comprehensive and high-throughput understanding of meat composition, nutritional value, and safety with a combination of biochemical and mechanical mechanisms. Overall, the techniques for lipidomics have been steadily progressing, particularly regarding the omics-data-mining and multivariate statistical analyses, whereby new efforts are contributed toward new algorithms of developed prediction models for identified lipid biomarkers. Untargeted MS-/NMR-based lipidomics gives molecular insight into meat origin/adulteration and microbial safety with more tentative lipid markers being screened out on a global scale, though additional targeted analytes (e.g., the lipolysis fate of PLs resulting from foodborne microbe bred in muscle) still require further validation in their adulteration detection. In addition, the exhaustive analysis of lipids and their alterations during meat production favors the selective design of processing methods for specific muscle matrices (e.g., irradiation and PEF). Many putative lipid biomarkers following computational approaches and possible metabolism pathways enriched by bioinformatics provide valuable suggestions on food safety and health concerns regarding their potential during the treatment with preservatives, fermentation, aging, and storage. However, challenges remain due to the complexity of meat lipidome, the nature of key intermediate lipid-metabolites, and their evaluation concerning the quality and nutritional value of the final product.

## Author Contributions

CL and BO-K performed literature review, analyzed and interpreted the data, and drafted the manuscript. CL, BO-K, and GJ reviewed the first draft and revised the manuscript accordingly. All authors contributed to the article and approved the final submitted version.

## Conflict of Interest

The authors declare that the research was conducted in the absence of any commercial or financial relationships that could be construed as a potential conflict of interest.

## Publisher’s Note

All claims expressed in this article are solely those of the authors and do not necessarily represent those of their affiliated organizations, or those of the publisher, the editors and the reviewers. Any product that may be evaluated in this article, or claim that may be made by its manufacturer, is not guaranteed or endorsed by the publisher.
